# microRNA-181c-5p promotes the formation of insulin-producing cells from human induced pluripotent stem cells by targeting smad7 and TGIF2

**DOI:** 10.1038/s41419-020-2668-9

**Published:** 2020-06-15

**Authors:** Ning Li, Doukou Jiang, Qian He, Fei He, Yang Li, Chunyan Deng, Furong Li

**Affiliations:** 1 Translational Medicine Collaborative Innovation Center, Shenzhen People’s Hospital (The Second Clinical Medical College, Jinan University; The First Affiliated Hospital, Southern University of Science and Technology), Shenzhen, 518020 Guangdong China; 2 Shenzhen Cell Therapy Public Service Platform, Shenzhen People’s Hospital (The Second Clinical Medical College, Jinan University; The First Affiliated Hospital, Southern University of Science and Technology), Shenzhen, 518020 Guangdong China; 3 Shenzhen key Laboratory of Stem Cell Research and Clinical Transformation, Shenzhen People’s Hospital (The Second Clinical Medical College, Jinan University; The First Affiliated Hospital, Southern University of Science and Technology), Shenzhen, 518020 Guangdong China; 4 Integrated Chinese and Western Medicine Postdoctoral research station, Jinan University, Guangzhou, 510632 Guangdong China

**Keywords:** Stem-cell differentiation, Endocrine system and metabolic diseases

## Abstract

Generating insulin-producing cells (IPCs) from human pluripotent stem cells is a promising method for studying the molecular mechanism underlying pancreas development and a potential treatment source for type 1 diabetes. Previous studies have shown that miR-181c-5p is highly enriched in adult islets; however, its role in pancreatic β cell differentiation is poorly understood. In this study, we differentiated human induced pluripotent stem cells (hiPSCs) into IPCs in a stepwise process that recapitulated pancreas organogenesis and observed that miR-181c-5p continuously accumulated throughout the entire differentiation process. hiPSCs were transduced with lentiviral vectors containing human miR-181c-5p precursor, which significantly increased the endodermal markers SOX17, FOXA2, CXCR4 and GATA4 and pancreatic endocrine-specific gene expression, including PDX1, NKX6.1, MAFA and Insulin. miR-181c-5p overexpression exerted little effect on the efficiency of definitive endoderm, whereas it promoted the differentiation of pancreatic progenitors and IPCs, especially for NKX6.1-positive and insulin-positive cells differentiation. Transplanted these cells exhibit glucose-stimulated C-peptide secretion in vivo and protect mice from chemically induced diabetes. It was found that miR-181c-5p directly targets the 3′UTR of smad7 and TGIF2 mRNA, which are known to be endogenous repressors of TGF-β-smad2/3 signaling, to decrease their mRNA and protein levels. Furthermore, overexpressed miR-181c-5p led to an elevation of the smad2/3 phosphorylation levels in hiPSC-derived cells, while treatment with smad2/3 inhibitors following miR-181c-5p overexpression had opposite effects on IPC formation. These results suggest that miR-181c-5p is critically involved in pancreatic lineage commitment through direct repression of smad7 and TGIF2 and that it modulates TGF-β-smad2/3 signaling activation and increases the feasibility of using patient-specific hiPSCs for β cell replacement therapy for type 1 diabetes.

## Introduction

Type 1 diabetes (T1D) is caused by the autoimmune destruction of β cells in the islets of Langerhans in the pancreas, leading to insulin deficiency and hyperglycemia^1^. Currently, either the administration of exogenous insulin or islet transplantation is the mainstay of treatment for T1D. However, exogenous insulin replacement therapy fails to achieve tight glycemic control and is insufficient for preventing chronic complications, including retinopathy, cardiovascular pathology, kidney failure, diabetic foot, and neuropathy^2^. Islet transplantation can achieve superior glucose homeostasis, but it is limited by the scarcity of pancreatic donors and the necessity for life-long immunosuppression^2,3^. The generation of pancreatic β-like cells from human pluripotent stem cells (hPSCs), which include embryonic stem cells (hESCs) and induced pluripotent stem cells (hiPSCs), is a potential alternative source of insulin-producing cells (IPCs) for cell replacement therapy^4^.

Comprehensive knowledge of the signaling pathways and temporal transcription factor activation patterns during human and rodent pancreas organogenesis has accelerated the generation of IPCs from hPSCs^5–7^. Recently, several studies have shown significant advancement in the in vitro differentiation of hESCs and hiPSCs into a β cell phenotype using extrinsic protein factors and small molecules^8–10^. These cells were able to secrete human insulin in response to glucose challenge and ameliorate hyperglycemia after being transplanted in diabetic mice. However, to date, these protocols have yet to yield β-like cells with high efficiency, and the cells have an immature phenotype. Instead, hPSC-derived β-like cells were functionally restricted, either having low levels of insulin synthesis or lacking appropriate insulin release in response to high glucose, which makes them more reminiscent of fetal pancreatic β cells^8,11^. Therefore, improving the IPC differentiation efficiency and function is still an urgent demand that remains to be addressed.

Recently, accumulated evidence has revealed that microRNAs (miRNAs) play a critical role in embryonic development^12^, cell fate decision^13^, metabolism^14^, pancreatic development^15^, β cell differentiation^16,17^, insulin secretion^17^, and glucose homeostasis^18^. Conditional deletion of Dicer, an essential enzyme for miRNA processing, in the developing pancreas results in gross defects in all pancreatic lineages, especially the insulin-producing β-cells^19^. In fact, a number of miRNAs are reported to be important regulators of β cell differentiation and function, including miR-375^17^, miR-26^20,21^, miR-24, miR-148^20^, miR-30d^22,23^, miR-21^24^, let-7^25,26^, miR-34a and miR-34c^27,28^, miR-7^29^, miR-145^30^, and miR-9^31,32^. The miR-181 family contains four miRNAs (miR-181a/b/c/d) in which the genomic sequences encoding miR-181c and miR-181d are located on human chromosome 19. Previous studies demonstrated that the overexpression of miR-181c promoted the differentiation of hESCs^33^, which is upregulated in hPSC-derived retinal pigment epithelium and cardiomyocytes^34,35^. However, the function of miR-181c in pancreatic β cell differentiation has not been fully elucidated.

The goal of the present study was to gain further insight into the mechanisms underlying the differentiation of hiPSCs toward a β-cell phenotype and particularly to identify miR-181c-5p and its targets involved in IPCs formation. We demonstrate a novel approach in which miR-181c-5p overexpression contributes to inducing hiPSCs differentiation into IPCs. Furthermore, smad7 and TGIF2 were post-transcriptionally regulated and were directly targeted by miR-181c-5p, which repressed the 3′ untranslated region (3′UTR) of smad7 and TGIF2. We also found that inhibition of smad2/3 phosphorylation partly rescued the effects of miR-181c-5p overexpression and resisted hiPSCs differentiation cues. These findings present a step towards cell replacement therapy for T1D by differentiation induction of hiPSCs into IPCs through miRNA overexpression.

## Materials and methods

### hiPSCs culture and differentiation

**Fig. 1:**
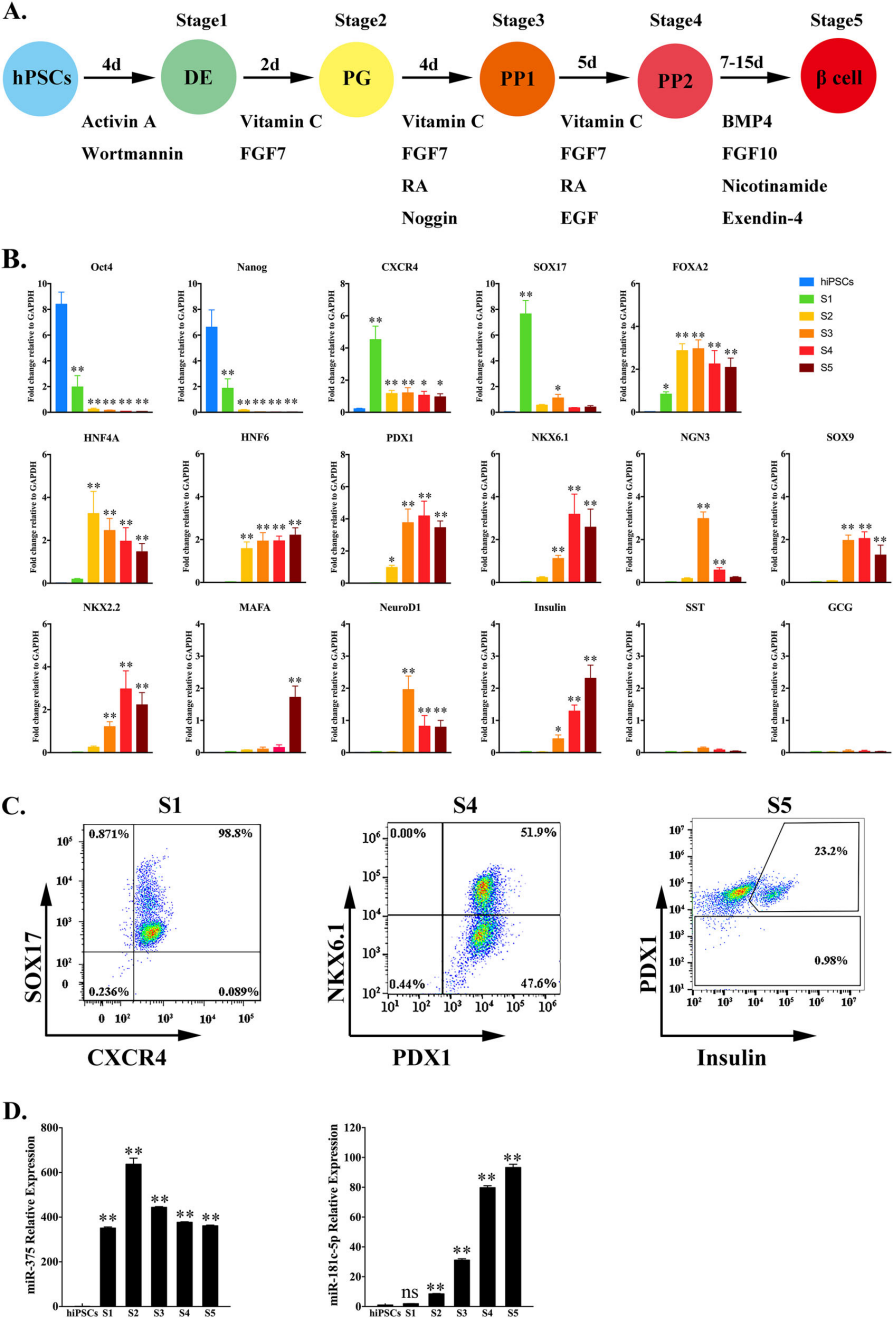
Pancreatic β-like cell differentiation of hiPSCs using a five-stage protocol. **a** Schematic strategy for the differentiation of hiPSCs into pancreatic β-like cells. DE, definitive endoderm; PG, primitive gut tube; PP, pancreatic progenitor; RA, retinoic acid. **b** Gene expression profile of hiPSC-derived cells at the S1-S5 stages of differentiation compared with hiPSCs. The data are expressed as the mean ± SD of three independent experiments (*n* = 6). ^*^p ≤ 0.05, ^**^p ≤ 0.001, relative to the hiPSCs control. **c** Representative flow cytometry plots illustrating the protein expression of CXCR4 and SOX17 (S1), PDX1 and NKX6.1 (S4), and PDX1 and insulin (S5) in populations of hiPSC-derived cells. **d** The dynamic expression levels of miR-375 and miR-181c-5p during the formation of IPCs. The data are expressed as the mean ± SD of three independent experiments (*n* = 8). ^**^p ≤ 0.001, relative to the hiPSCs control. ns, not statistically significant. S1, stage 1; S2, stage 2; S3, stage 3; S4, stage 4; S5, stage 5.

The iPS cell line was established by the Guangzhou Institutes of Biomedicine and Health, Chinese Academy of Sciences, and prepared from reprogramming of a 34-year-old woman’s dermal fibroblast cells. hiPSCs were cultured using mTeSR1 medium (Stem Cell Technology, Vancouver, BC, Canada) on Matrigel (BD Biosciences, San Jose, CA, USA)-coated culture plates. The five-stage procedure (Fig. 1a) for in vitro differentiation of hiPSCs into IPCs was slightly modified from previously published protocols^8,9,36^.

Stage 1: formation of definitive endoderm (DE). The hiPSCs were dissociated into small clumps by Dispase II (Gibco, USA) and were collected by sedimentation. The dissociated colonies were plated on Matrigel (1:50)-coated plates for attachment and incubated with DMEM/F12 (GluMax, Gibco) supplemented with 0.2% BSA (Sigma-Aldrich, USA), 100 ng/ml activin A (Peprotech, USA), 1 μM wortmannin (Selleck, USA), 1:100 B27 (Gibco), and 1:200 N2 (Gibco) for 4 days.

Stage 2: formation of primitive gut tube (PG). DE cells were rinsed with 1× DPBS (without Mg^2+^ and Ca^2+^, Gibco) and then cultured in DMEM/F12 (GluMax) with 0.5% BSA, 0.25 mM Vitamin C (Sigma-Aldrich), 1:200 ITS-X (Gibco), and 50 ng/ml FGF7 (Peprotech) for 2 days.

Stage 3: formation of pancreatic progenitor cells (PP1). PG cells were rinsed with 1× DPBS and then exposed to IMDM/F12 (GluMax, 1:1, Gibco) medium further supplemented with 2% BSA, 0.25 mM Vitamin C, 1:200 ITS-X, 1:100 B27, 2 μM retinoic acid (RA, Sigma-Aldrich), 50 ng/ml FGF7, and 100 ng/ml Noggin (Peprotech) for 4 days.

Stage 4: formation of pancreatic progenitor cells (PP2). PP1 cells were rinsed with 1× DPBS and then exposed to DMEM (high glucose, Gibco) with 2% BAS, 0.25 mM Vitamin C, 1:100 ITS-X, 1:100 N2, 100 nM RA, 20 ng/ml FGF7, and 50 ng/ml EGF (Peprotech) for 5 days.

Stage 5: formation of IPCs. PP2 cells were rinsed with 1× DPBS and then exposed to DMEM/F12 (GluMax) with 1:200 ITS-X, 10 ng/ml BMP4 (Peprotech), 10 ng/ml FGF10 (Peprotech), 10 mM Nicotinamide (Sigma-Aldrich), and 50 ng/ml Exendin-4 (Sigma-Aldrich) for 7–15 days.

### Quantitative real-time PCR for mRNA and miRNA

Total RNA was extracted from samples using TRI-Reagent (Sigma-Aldrich) according to the manufacturer’s instructions. To assess mRNA expression (Supplementary Table S1), reverse transcription-PCR was performed using an RT reagent kit with gDNA eraser (TaKaRa, Japan). Quantitative real-time PCR was performed on an ABI 7500 system (Applied Biosystems, USA) using SYBR Green PCR Master Mix (Roche Diagnostics, USA). A standard curve was derived from the serial dilutions. Gene expression levels were normalized to the internal control (GAPDH).

To profile miR-375 and miR-181c-5p expression, the polyadenylation, reverse transcription and TaqMan-based quantitative PCR procedure were conducted exactly according to the S-Poly(T) Plus protocol^37^. miRNAs with a cycle threshold (Ct) value less than 35 in the panel were included in the data analysis, and the fold change was normalized to spiked-in cel-miR-54-5p. Each example was run in triplicate. All sequences, primers and probes are listed in the Supplementary Table S1 and Supplementary Table S2. The relative expression levels of miRNA were calculated according to the formula 2^-∆∆Ct^.

### Glucose-stimulated insulin secretion

Human islets (~20–50 islets) and hiPSC-derived cells (1~2 × 10^6^) were rinsed twice with Krebs buffer (Sigma-Aldrich) and pre-incubated in Krebs buffer for 1 h to remove residual insulin. Cells were then incubated in Krebs buffer containing 2.8 mM glucose for 1 h, followed by incubation in Krebs buffer containing 16.7 mM glucose. Supernatant samples were collected after each incubation period, centrifuged to remove cell debris and frozen at −80 °C. Human insulin and C-peptide released into the medium were measured by an ELISA kit (R&D) according to the manufacturer’s instructions. Total protein contents were measured using a BCA protein assay kit (Pierce Biotechnology, USA), and insulin/C-peptide levels were normalized to protein levels. Written informed consent was obtained from all participants, and the use of human samples was approved by the Ethics Committees of the Second Clinical Medical College of Jinan University.

### Hormone content

To measure the intracellular hormone content, the cells were washed three times with DPBS, and harvested in RIPA buffer [1% Triton X-100, 150 mM NaCl, 50 mM Tris-HCl (pH 7.4), 1 mM phenylmethylsulfonylfluoride (PMSF), 5 mg/ml aprotinin, 5 mg/ml leupeptin, 0.1 mM NaF, 10 μM E-64, and 5 mg/ml pepstatin A]. Cell extracts were sonicated and centrifuged at 14,000 *g* at 4 °C, and the proinsulin, insulin and C-peptide contents were quantified using ELISA kits (R&D). Cells were lysed in acid ethanol solution for total DNA, and the hormone value was normalized to the total cellular DNA content from the respective lysates.

### Luciferase assays

293T cells were cultured in 24-well plates, transfected with different reporter vectors (pmirGLO-control, pmirGLO-3′UTR smad7, pmirGLO-3′UTR smad7 MUT1, pmirGLO-3′UTR smad7 MUT2, pmirGLO-3′UTR TGIF2, pmirGLO-3′UTR TGIF2 MUT1, and pmirGLO-3′UTR TGIF2 MUT2) and co-transfected with miRNA-control or miR-181c-5p mimic (150 nM) by using Lipofectamine 3000 (Life Technologies, USA). All constructs were confirmed by DNA sequencing. Relative firefly luciferase activity was measured 24 h after transfection using the Dual-Luciferase Reporter Assay Kit (Promega, USA). Luciferase activity was normalized to Renilla luciferase activity. All transfections were repeated independently at least three times.

### Statistical analysis

All experiments were repeated independently in three or more times under identical conditions. Data are presented as the mean ± SD. mRNA, miRNA, hormone secretion, and luciferase data analysis were calculated by using one-way or two-way ANOVA test (nonparametric tests); in vivo data was calculated by using two-tailed Student’s *t*-test (nonparametric tests). Calculations were conducted using GraphPad 8.0 (GraphPad Software, Inc., San Diego, California, USA). *p* ≤ 0.05 was considered statistically significant.

## Results

### Generation of pancreatic β-like cells in vitro

To investigate the function of specific miRNAs in hPSC differentiation into pancreatic β cells, we developed a five-step protocol for differentiation of hiPSCs into pancreatic β-like cells (Fig. 1a). With the optimized differentiation protocol, hiPSC-derived stage 1 cells maintained robust co-expression of endoderm genes, such as SOX17, FOXA2, and CXCR4 (Fig. 1b, c). In addition, the pluripotency genes Oct4 and Nanog significantly declined and then disappeared at stages 4 and 5 (Fig. 1b). In stage 2, we observed a considerable upregulation of the gut tube marker HNF4A and slightly increased expression of HNF6 and PDX1. Simultaneously, expression of the DE markers SOX17 and CXCR4 was markedly reduced; however, FOXA2 continued to be expressed in stage 2–5 cells, demonstrating their endodermal origin (Fig. 1b). During stages 3–4, the primitive gut tube cells were exposed to retinoic acid (RA), Noggin and EGF. These cells rapidly began to express high levels of PDX1 and NKX6.1 (Fig. 1b, c), while increasing the co-expression of NGN3, NKX2.2, and NeuroD1. Expression of this combination of genes is indicative of pancreatic endoderm and endocrine precursors. Moreover, NGN3 mRNA expression was transiently induced in the stage 3 population along with its downstream target expression, including NXK6.1, NKX2.2, and NeuroD1 (Fig. 1b). In stage 5, pancreatic hormone insulin and its transcription factor MAFA were robustly expressed, but not SST and GCG (Fig. 1b, c). Although these cells expressed SOX9, we noted a significant drop in SOX9 mRNA expression in stage 5, consistent with previous results suggesting loss of SOX9 expression during maturation of β cell precursors^38^. Overall, the expression dynamics suggested that the stepwise differentiation protocol used in this study recapitulated key developmental stages in human pancreatic β cell specialization.

### miR-181c-5p promotes the differentiation of hiPSCs into IPCs

To assess pancreatic β-cell-specific miRNAs, as a positive marker, we observed the upregulation of miR-375 (the most abundant islet miRNA) from the DE to PG stage, followed by a slight decrease in the last stage of differentiation (Fig. 1d). Notably, the expression pattern of miR-181c-5p was low in hiPSCs and DE cells, followed by a rapid increase during primitive gut tube formation, and continuously accumulated throughout the entire differentiation process (Fig. 1d), consistent with previous results showing upregulation of miR-181c-5p expression during pancreatic lineage differentiation^39,40^.

**Fig. 2:**
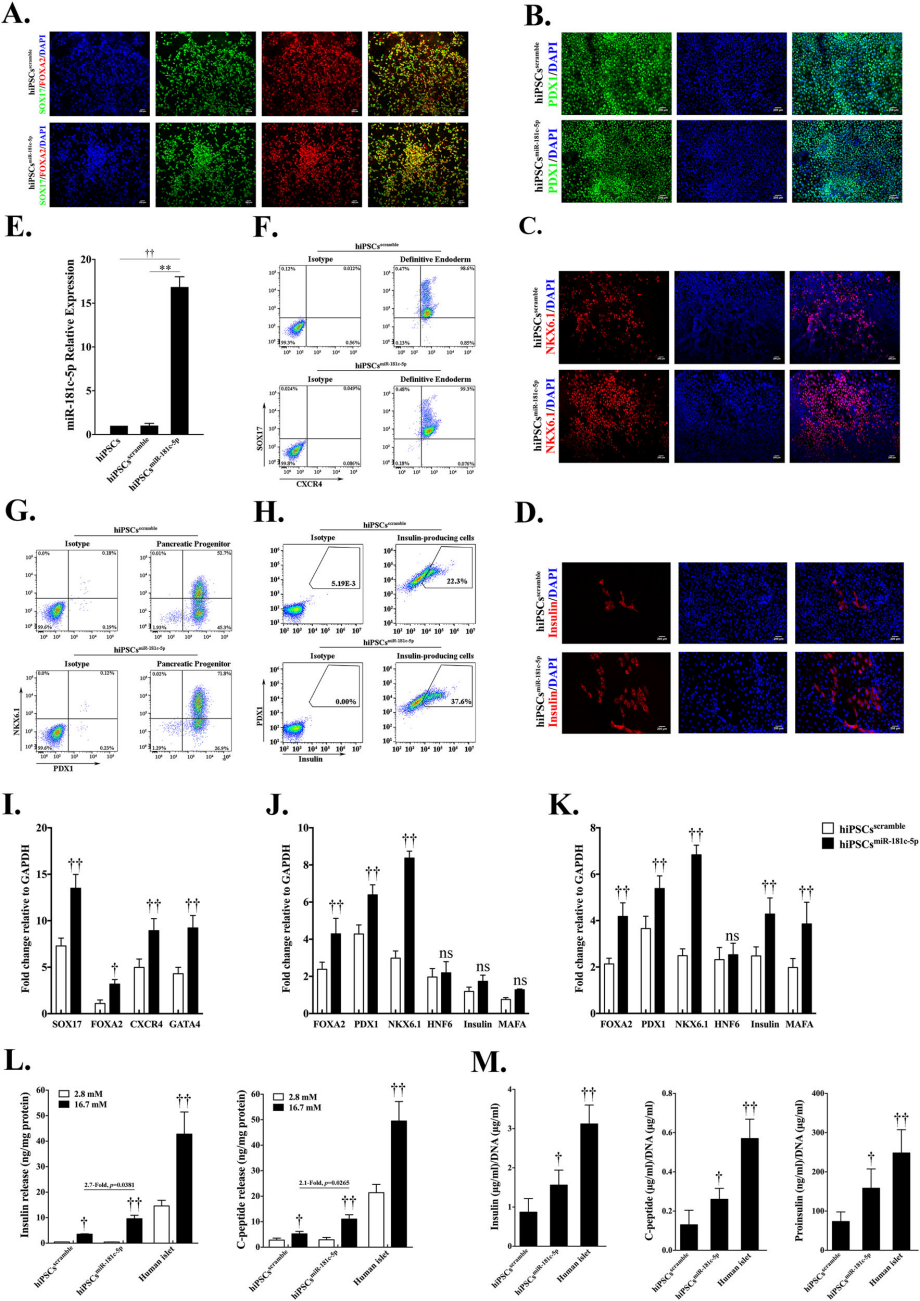
Overexpression of miR-181c-5p promotes the expansion of hiPSC-derived pancreatic β-like cells. **a** Immunofluorescence staining of definitive endoderm images for SOX17 and FOXA2 at day 4 (S1) (original magnification, ×200). **b**, **c** Immunofluorescence staining of pancreatic progenitor cell images for PDX1 or NKX6.1 at day 15 (S4) (original magnification, ×200). **d** Immunofluorescence staining of pancreatic β-like cells images for insulin at day 24 (S5) (original magnification, ×200). **e** The expression of miR-181c-5p in hiPSCs that were infected with miR-181c-5p or negative control lentivirus or not infected was monitored by qRT-PCR. The data are expressed as the mean ± SD of three independent experiments (*n* = 7). ^**^p ≤ 0.001, relative to the negative control (hiPSCs^scramble^). ^††^*p* ≤ 0.001, relative to the hiPSCs control. **f** Representative flow cytometry dot plots and population percentages of cells stained for SOX17 and CXCR4. **g** Representative flow cytometry dot plots and population percentages of cells stained for PDX1 and NKX6.1. **h** Representative flow cytometry dot plots and population percentages of cells stained for PDX1 and insulin. **i**–**k** Real-time PCR analysis of the selected markers SOX17, FOXA2, CXCR4, GATA4, PDX1, NKX6.1, HNF6, insulin and MAFA following miR-181c-5p overexpression in hiPSCs. The data are expressed as the mean ± SD of three independent experiments (*n* = 11). ^†^*p* ≤ 0.05, ^††^*p* ≤ 0.001, relative to the negative control (hiPSCs^scramble^). ns, not statistically significant. **l** Glucose-stimulated insulin secretion (GSIS) of human islets and hiPSC-derived β-like cells at day 24 (*n* = 6). The data are expressed as the mean ± SD of three independent experiments. ^††^*p* ≤ 0.001, relative to the 2.8 mM group. **m** Hormone content relative to DNA content for human islets and hiPSC-derived β-like cells (*n* = 7). The data are expressed as the mean ± SD of three independent experiments. ^†^*p* ≤ 0.05, ^††^*p* ≤ 0.001, relative to the negative control (hiPSCs^scramble^).

**Fig. 3:**
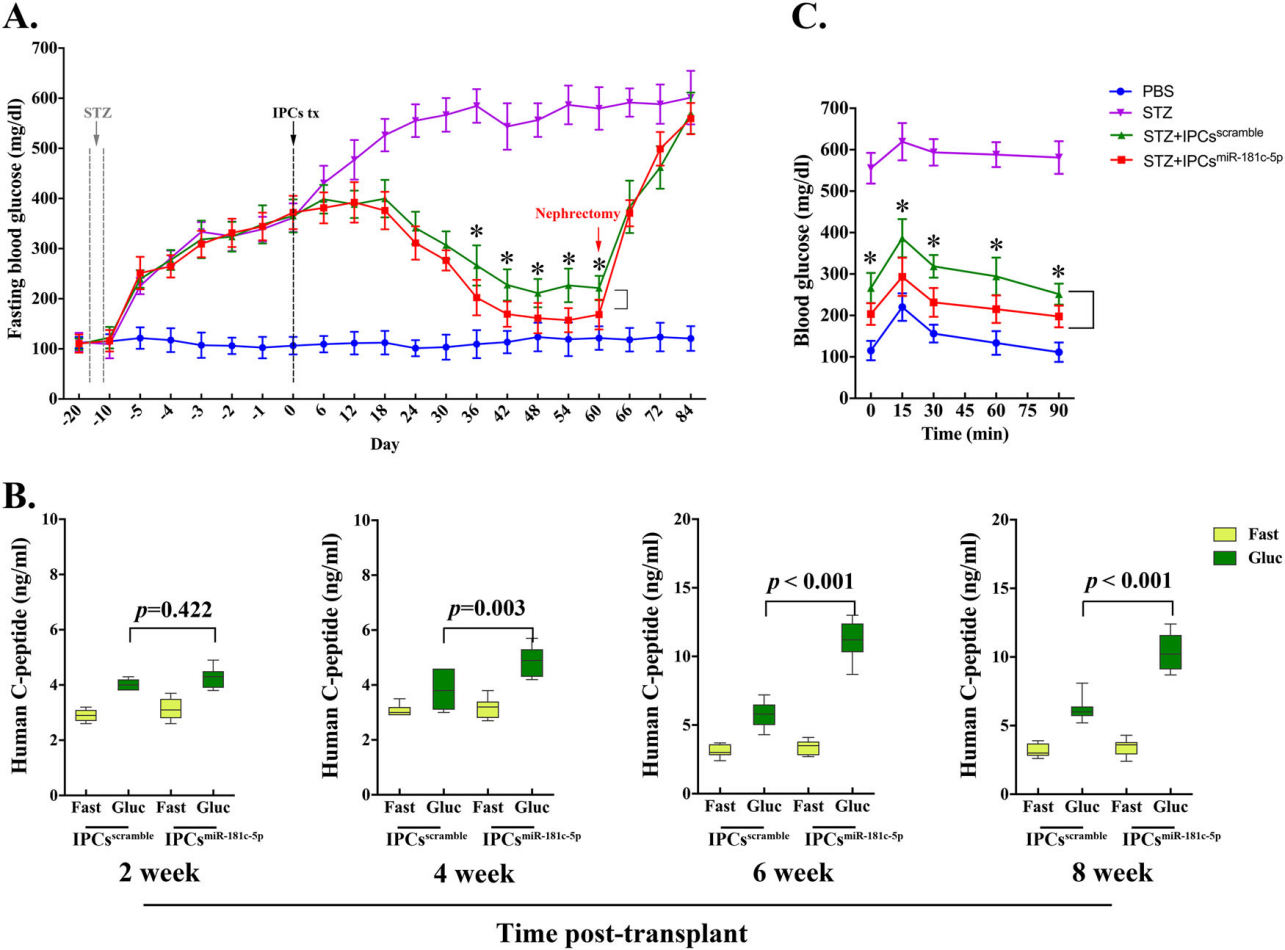
Transplanted insulin-producing cells ameliorate hyperglycemia and remain glucose responsive in diabetic mice. Mice were treated with the mouse-specific beta-cell toxin streptozotocin (STZ, 35 mg/kg via intraperitoneal injection for 5 days) to ablate endogenous beta cells. Insulin-producing cells (~1 × 10^7^ cells/animal) were transplanted under the kidney capsule of diabetic mice. **a** Fast blood glucose was assessed every 6 days throughout the study and a survival nephrectomy was performed on all mice on day 60 to remove the engrafted kidney (indicated by red arrow). Unilateral nephrectomy of IPCs graft-bearing mice resulted in a rapid rise in fast blood glucose levels, directly demonstrating euglycemic control due to IPCs grafts after STZ treatment in these mice (*n* = 7). IPCs^scramble^, control hiPSCs-derived insulin-prodcuing cells. IPCs^miR-181c-5p^, miR-181c-5p overexpressed hiPSCs-derived insulin-prodcuing cells. ^*^p ≤ 0.05, STZ + IPCs^miR-181c-5p^ group compares with STZ + IPCs^scramble^ group. **b** ELISA analysis of serum from fasted and glucose-challenged (3 g/kg) mice post transplantation. IPCs graft-bearing mice exhibit significant higher levels of circulating human C-peptide in serum after glucose challenge, indicating that transplanted IPCs cells remain functional in vivo. Mice transplanted with miR-181c-5p overexpressed hiPSCs-derived IPCs exhibit significant higher levels of human C-peptide at 4-week, 6-week, and 8-week post transplantation. **c** Blood glucose levels were measured during an intraperitoneal injection glucose tolerance test at 8-week post transplantation. ^*^p ≤ 0.05, STZ + IPCs^miR-181c-5p^ group compares with STZ + IPCs^scramble^ group.

To explore the role of miR-181c-5p in the differentiation of IPCs from hiPSCs, we overexpressed miR-181c-5p in hiPSCs using a lentivirus system (Fig. 2e). In stage 1, there was no significant change in SOX17 and FOXA2 co-expression cells in both groups (Fig. 2a), and flow cytometry data confirmed that overexpressed miR-181c-5p exerted little effect on the population of DE cells (Fig. 2f). However, increased mRNA levels of endodermal marker genes (SOX17, FOXA2, CXCR4, and GATA4) were found in the miR-181c-5p overexpression group compared with the control group (Fig. 2i). During PP cell differentiation, the population of PDX1-positive cells was observed to be robust (Fig. 2b, g). Interestingly, miR-181c-5p overexpression was found to be able to significantly increase NKX6.1 expression (Fig. 2j) and enhance the efficiency of NKX6.1-positive cells (Fig. 2c, g). Furthermore, no significant difference was observed in the expression profile of HNF6, insulin and MAFA (Fig. 2j). In the final stage, the efficiency of IPC differentiation from PP cells was higher with miR-181c-5p overexpression than that from control cells (Fig. 2d, h). In line with the elevated expression of insulin, we also detected a strong induction of the insulin transcriptional activator MAFA (Fig. 2k). Cells from all experimental groups and adult human islets were subjected to a glucose-stimulated insulin secretion test (GSIS) to measure their ability to respond to elevated glucose concentrations at day 24. When challenged with 16.7 mM glucose, a significant increase in hormone secretion was observed (Fig. 2l) in a manner comparable to that of adult human islet cells. As expected, insulin and C-peptide release by the miR-181c-5p overexpression group was approximately two-fold higher than that by the control group. Furthermore, the hormone content per μg of DNA in the miR-181c-5p overexpression group was 1.56 ± 0.38 μg (insulin), 0.26 ± 0.06 μg (C-peptide), 158.39 ± 49.04 ng (proinsulin). These values were significantly higher with respect to the control group: 0.87 ± 0.34 μg, 0.13 ± 0.07 μg, and 73.45 ± 24.29 ng, respectively (Fig. 2m). Considering these major improvements, we decided to evaluate their in vivo function by short-term transplanting IPCs (approximately 10 million) under the kidney capsule of mice with STZ-induced diabetes. As expected, mice that received grafts exhibited low levels of human C-peptide secretion upon fasting, followed by a marked increase after glucose challenge (Fig. 3b). IPCs graft-bearing mice exhibited significantly reduced blood glucose levels at all time points analyzed when compared with STZ-control animals (Fig. 3a). At 36 d post-transplant, fasting blood glucose levels of miR-181c-5p-overexpressing group were significantly lower than scramble-control group. Mice transplanted with miR-181c-5p overexpressed hiPSCs-derived IPCs exhibited significant higher levels of human C-peptide at 4-week, 6-week and 8-week post transplantation (Fig. 3b). By 8 weeks post-transplant, miR-181c-5p-overexpressing group exhibited significant lower levels of blood glucose after glucose challenge, when compared with the scramble-control group (Fig. 3c), indicating that transplanted IPCs cells remain functional in vivo. Furthermore, IPCs graft-bearing mice return to hyperglycemia were observed after graft removal (Fig. 3a). Taken together, these data highlight the function of miR-181c-5p in the differentiation of IPCs and demonstrate that miR-181c-5p-overexpressing hiPSCs represent a step towards committed β-like cells.

### TGIF2 and smad7 are direct targets of miR-181c-5p

**Fig. 4:**
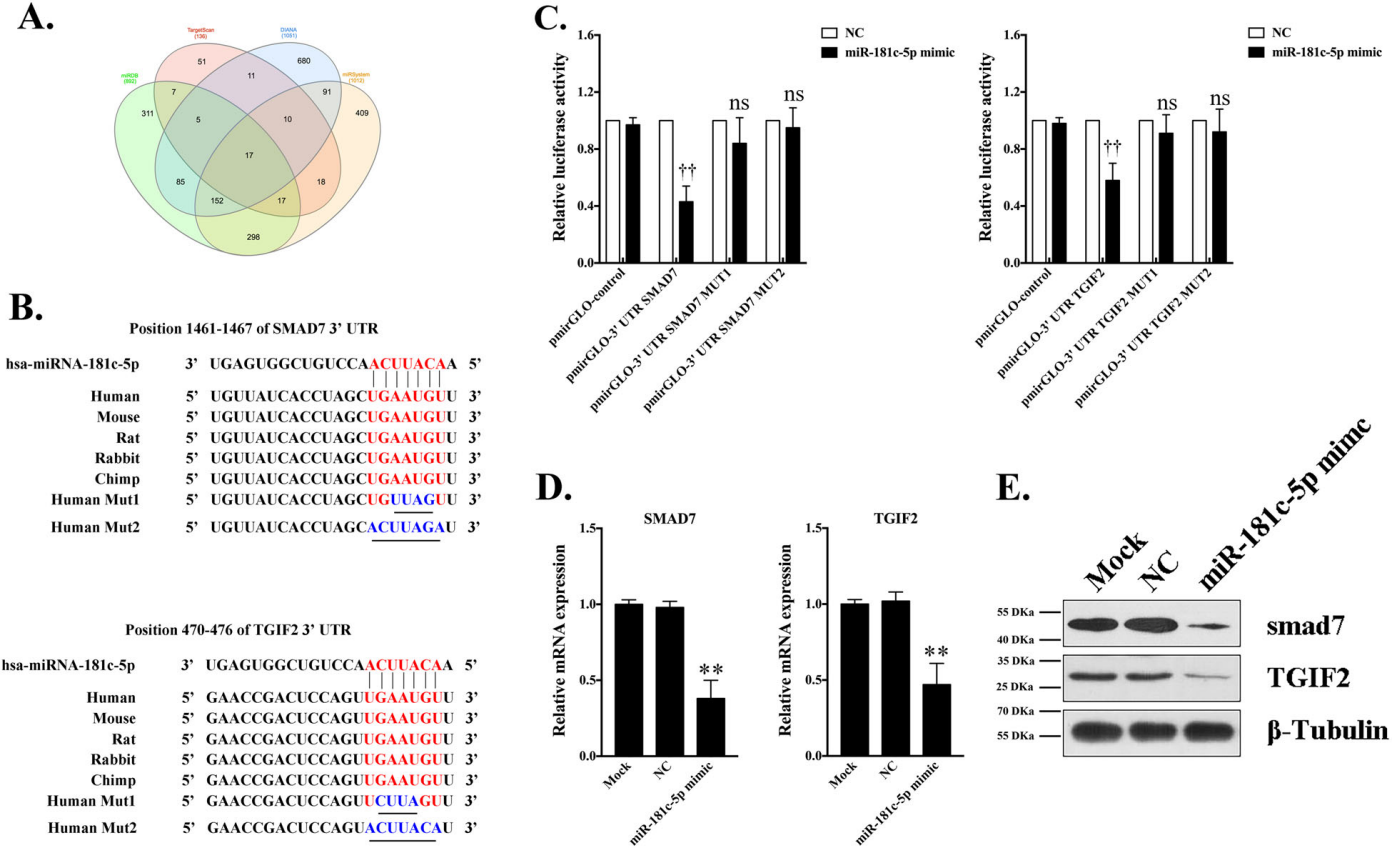
Candidate target analysis of miR-181c-5p. Targets of miR-181c-5p were predicted using the TargetScan, miRDB, DIANA, and miRSystem tools, and analyzed using Venn tools. Seventeen direct target genes of miR-181c-5p that may be involved in the formation and maturation of IPCs were found in the Venn diagram (**a**). Combining previous reports, smad7 and TGIF2 were selected to test the regulatory relationship after miR-181c-5p overexpression in hiPSCs. The location and sequences of the miR-181c-5p target site in the 3′UTR of human smad7 and TGIF2 (**b**). The sequence of human miR-181c-5p is indicated, along with mutations introduced in the target site (underlined nucleotides) for generating the mutated reporter construct (**b**). 293T cells were co-transfected with luciferase 3’UTR reporter vectors containing 3’UTR fragments of wild-type smad7/TGIF2 and mutated smad7/TGIF2 in the presence of miR-181c-5p mimic or negative control. Luciferase activity was assayed 24 h after transfection (**c**, *n* = 8). All luciferase values were normalized to Renilla luciferase. The data are expressed as the mean ± SD of three independent experiments. ^††^*p* ≤ 0.001, relative to the negative control (NC). ns, not statistically significant. **d**, **e** Real-time PCR and western blot analysis of mRNA and proteins expressed by gene targets of miR-181c-5p following miR-181c-5p overexpression in 293T cells. The data are expressed as the mean ± SD of three independent experiments (*n* = 8). ^**^p ≤ 0.001, relative to the hiPSCs control (Mock).

To elucidate the mechanism of miR-181c-5p in the formation of IPCs, the targets of miR-181c-5p were predicted using TargetScan, miRDB, DIANA and miRSystem and analyzed using the Venn tool (Fig. 4a). Because TGF-β/smad signaling is an important determinant of pancreas development, smad7 and TGIF2, which serve as endogenous inhibitors, were selected as candidate targets. The predicted miR-181c-5p binding site in the 3′UTR of smad7 and TGIF2 mRNA appeared to be phylogenetically maintained (Fig. 4b). This outcome suggested that the function of miR-181c-5p as a potential regulator of smad7 and TGIF2 has been conserved in humans, mice, rats, rabbits and chimpanzees. To investigate whether miR-181c-5p affects smad7 and TGIF2, synthetic miR-181c-5p mimics or miRNA-control were transfected into 293T cells. miR-181c-5p overexpression attenuated smad7 and TGIF2 mRNA and protein expression (Fig. 4d, e), indicating that miR-181c-5p acts as a post-transcriptional repressor. To evaluate whether the predicted miR-181c-5p target sites in the 3′UTR of smad7 and TGIF2 mRNA were directly involved in the miR-181c-5p-induced reduction of smad7 and TGIF2 expression, a plasmid driving the putative 3′UTR target site, a mutation or nonsense sequence was cloned downstream of a luciferase reporter gene and co-transfected with miRNA-control or miR-181c-5p mimic into 293T cells. The luciferase activity of cells transfected with pmirGLO-3′UTR smad7/pmirGLO-3′UTR TGIF2 and miR-181c-5p mimic was dramatically decreased (Fig. 4c). However, when the predicted binding sites were mutated, the luciferase activity was efficiently restored to control levels. Collectively, our data argued for a direct interaction between miR-181c-5p and smad7/TGIF2 mRNA.

### miR-181c-5p promotes the differentiation of IPCs by modulating the TGF-β-smad2/3 pathway

**Fig. 5:**
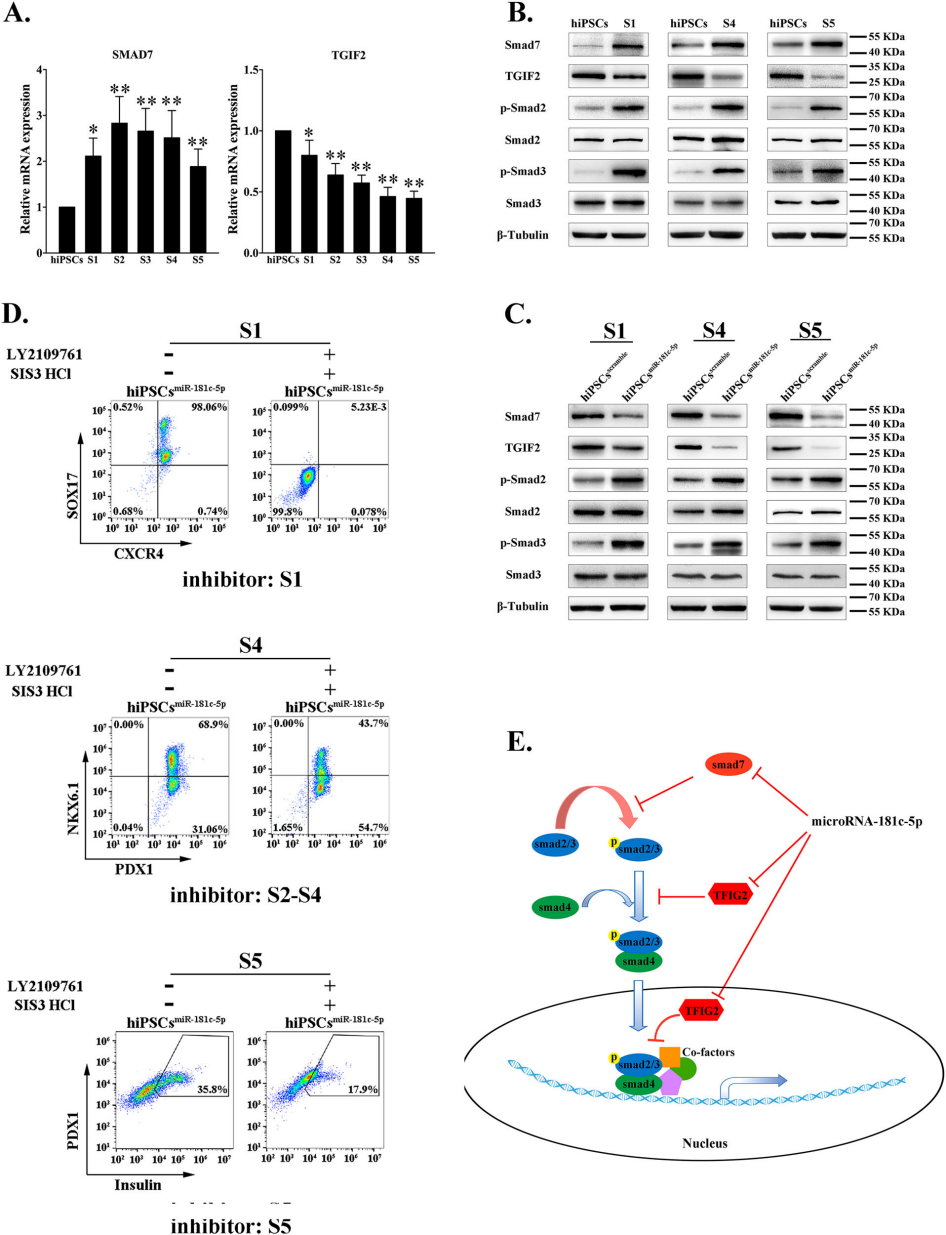
miR-181c-5p leads to hiPSCs differentiation into IPCs through negative regulation of smad7 and TGIF2. **a**, **b** The dynamic expression levels of smad7 and TGIF2 at the mRNA and protein levels were quantified by real-time PCR and western blot, respectively. hiPSCs were infected with miR-181c-5p or negative control lentivirus and then induced into IPCs. The data are expressed as the mean ± SD of three independent experiments (*n* = 7). ^*^p ≤ 0.05, ^**^p ≤ 0.001, relative to the hiPSCs control. **c** Endogenous expression levels of smad7, TGIF2, and phosphorylation-smad2/3 were analyzed by western blot. **d** The effect of phosphorylation-smad2 and phosphorylation-smad3 inhibitors on differentiated hiPSCs was evaluated by flow cytometry. Inhibitor: S1, inhibitors only used in stage 1; Inhibitor: S2-S4, inhibitors used from stage 2 to stage 4; Inhibitor: S5, inhibitors only used in stage 5. LY2109761, phosphorylation-smad2 inhibitor. SIS3 HCl, phosphorylation-smad3 inhibitor. **e** Proposed mechanism for the regulation of hiPSCs differentiation into IPCs by microRNA-181c-5p.

To further verify the promoting mechanism of miR-181c-5p in the differentiation of IPCs, we identified the dynamic expression of smad7 and TGIF2 during differentiation. TGIF2 mRNA was attenuated throughout the entire differentiation process (Fig. 5a). In contrast, smad7 mRNA markedly increased in differentiated cells. Western blot analysis confirmed this opposite expression pattern (Fig. 5b). Additionally, miR-181c-5p overexpression significantly inhibited the protein levels of TGIF2 and smad7 during differentiation (Fig. 5c). TGIF2 and smad7 are known as TGF-β-smad2/3 pathway inhibitors; thus, we evaluated whether smad2/3 activity is affected by miR-181c-5p and whether smad2/3 mediates the miR-181c-5p-induced IPC differentiation. Our results showed that smad2/3 activation dramatically increased in differentiated cells with respect to undifferentiated hiPSCs (Fig. 5b, stages 1, 4, and 5). Furthermore, miR-181c-5p-overexpressing cells displayed a higher level of smad2/3 phosphorylation compared with scramble lentivirus-infected cells (Fig. 5c). To directly validate that an augmentation of smad2/3 activity is involved in IPC differentiation, we simultaneously employed the smad2 inhibitor LY2109761 and the smad3 inhibitor SIS3. After treatment of differentiated cells for the indicated times, inhibition of smad2/3 phosphorylation (data not shown), accompanied by significantly decreased efficiency of DE, pancreatic progenitor and IPC differentiation was observed in the background of miR-181c-5p overexpression (Fig. 5d). Taken together, these results suggest that miR-181c-5p is critically involved in the fate decision of hiPSCs and pancreas lineage commitment through direct repression of smad7 and TGIF2 and consequently activation of smad2/3 signaling (Fig. 5e).

## Discussion

Developing alternative ways to generate pancreatic β cells is a promising approach for cell therapy treatments for T1D. Induced pluripotent stem cells (iPSCs) provide an alternative cell source for regenerative medicine in diabetes because they are characterized by unlimited self-renewal and can be derived from diabetic patients, which are eventually used to generate autologous therapeutic β cells for transplantation^41^. Recent advances have been made to obtain functional β-like cells from pluripotent cells^8,9^; however, to date, these protocols are suboptimal and inefficient and the differentiated cells have an immature phenotype. Within this framework, the biochemical mechanisms that regulate pancreatic β cells differentiation, especially the modification of gene expression by miRNAs, have recently received increased attention.

In this study, we found that miR-181c-5p was progressively upregulated during the formation of IPCs derived from hiPSCs. Consistently, miR-181c is highly enriched in the late stage of hESCs differentiation, in the fetal pancreas and in human adult islets^39,40^. Furthermore, despite the common developmental origin shared by the two tissues, comparison of the miRNAs profiles between liver and pancreas revealed that miR-181c-5p was differentially expressed and was upregulated in pancreatic islets while downregulated in hepatocytes^42^. Therefore, we hypothesized that miR-181c-5p may play pancreatic-specific functions, and investigating the mechanism of miR-181c-5p in the formation of pancreatic β-like cells and adjusting the differentiation methods are highly significant for the regeneration of functional β cells.

It has been previously demonstrated that the TGF-β-smad2/3 signaling pathway is involved in the specification of embryonic pancreas^43^. miR-181c/d is regulated directly by the TGF-β-smad2/3 pathway in mouse ES cells and early embryos^44^. Furthermore, miR-181c promotes Th17 cell differentiation^45^ and inhibits neuroblastoma cell proliferation^46^ by targeting smad7. Smad7 is an inhibitory smad (I-SMAD) and interacts with activated TGF-β type I receptor, therefore blocking the phosphorylation and activation of smad2/3^47^. Our data indicated that smad7 was significantly increased during the IPC differentiation process. While smad7 is induced by activin or EGF, it also plays a role in the negative feedback of TGF-β-smad2/3 signaling. In addition to the alteration of this pathway, TGIF2 (homeodomain protein TG-interacting factor 2), a candidate target of miR-181c-5p, exerts cooperative functions with smad7. Activation of Ras protein stabilizes the smad repressor TGIF2, causing smad signaling transduction failure and transcriptional repression of smad4^48,49^. In this context, overexpression of miR-181c-5p allows for the increased expression of those genes involved in the formation and maturation of IPCs; on the other hand, the inhibition of smad7 and TGIF2 facilitates the phosphorylation of smad2/3, thus favoring the pancreatic β cell phenotype specification, especially for NKX6.1-positive and insulin-positive cell differentiation. Furthermore, we decided to evaluate their in vivo function by transplanting differentiated cells under the kidney capsule of immunocompromised mice. The data showed, in miR-181c-5p overexpression group, both the glucose-stimulated human C-peptide secretion increased and the fasting blood glucose levels decreased significantly comparing to the control group. In addition, although the luciferase analysis showed that smad7 and TGIF2 were direct targets of miR-181c-5p, the molecular mechanisms of the role of miR-181c-5p in regulating the activation of the TGF-β-smad2/3 pathway and downstream genes that are critically important in the specification of β cell fate and in the maturation of β cells should be further explored in the future.

In conclusion, this study unveils a miRNA-mediated mechanism for miR-181c-5p that regulates hiPSCs fate decisions and pancreatic β-like cell differentiation through direct repression of smad7 and TGIF2. The findings reported here present a new strategy for generating IPCs in vitro by miR-181c-5p overexpression and a potential source of IPCs for transplantation therapy of type 1 diabetes mellitus.

## Supplementary information

Supplementary Methods

Supplementary Table S1

Supplementary Table S2

Supplementary Table S3
