# Supplementary material for: microRNA-181c-5p promotes the formation of insulin-producing cells from human induced pluripotent stem cells by targeting smad7 and TGIF2
Source: Cell Death Dis. 2020 Jun 15;11(6):462. doi: 10.1038/s41419-020-2668-9 (PMC7295798; doi:10.1038/s41419-020-2668-9)
Supplement: Supplementary file 1 — Supplementary Methods [file 41419_2020_2668_MOESM1_ESM.docx]

**Supplementary Methods**

**Lentivirus-****mediated transfection**

The miR-181c-5p (MIMAT0000258) sequence was obtained from the miRBase database. miR-181c-5p lentiviral vector construction, identification, packaging and titration were completed by GeneChem (Shanghai, China). A lentiviral vector containing only green fluorescent protein (GFP) acted as a marker of infection efficiency. hiPSCs were infected with miR-181c-5p or negative control lentiviral vector (nonsensical sequence) at a multiplicity of infection (MOI) of 30 and then selected by 1 μg/ml puromycin.

**microRNA target gene prediction and Venn diagrams**

microRNA-181c-5p target gene prediction analysis was performed employing the online algorithm TargetScan (Release7.2, http://www.targetscan.org/vert_72/), DAVID Bioinformatics Resources 6.8 (https://david.ncifcrf.gov), miRSystem (http://mirsystem.cgm.ntu.edu.tw), and miRNAbase (http://www.mirbase.org/). Venn tools were used to demonstrate superposition relationships among the target genes of miR-181c-5p. Venn diagrams were generated using software available at <http://www.interactivenn.net/index.html> for further investigation of two pancreas development-related genes: smad7 and TGIF2. The sequences of miR-181c-5p binding sites in the 3’UTR of target genes were verified by TargetScan.

**Immunofluorescence analysis**

Cells were seeded into a 2-well chamber slide and fixed in 4% paraformaldehyde (PFA) in PBS for 20 min at room temperature (RT). The cells were permeabilized with 0.1% Triton X-100 in PBS for 10 min and blocked with 5% donkey serum for 1 h at RT and then incubated overnight with primary antibody at 4°C. The primary antibodies were mouse anti-human SOX17 (1:1000, #ab84990, Abcam, Shanghai, China), goat anti-human FOXA2 (1:200, AF2400, R&D, USA), rabbit anti-human PDX1 (1:1000, #ab47267, Abcam), mouse anti-human NXK6.1 (1:50, F55A12, DSHB, USA), and guinea pig anti-human insulin (1:200, ab7842, Abcam). Alexa 488-conjugated anti-mouse or rabbit IgG; Alexa 594-conjugated anti-goat, rabbit or mouse IgG; and Alexa 568-conjugated anti-guinea pig IgG (1:1000) were used as the secondary antibodies for 1 h at RT. The nuclei were stained with DAPI (1 μg/mL). After sequential excitation, images of the same cell were captured using the Leica DMi8 system and analyzed using ImageJ2x software.

**Flow cytometry**

Differentiated cells were dispersed into single-cell suspensions by incubation in TrypLE Express (Gibco) at 37°C. After washing twice with DPBS, the cells were fixed and permeabilized with Fixation/Permeabilization solution (BD Biosciences, USA) for 30 min at RT and incubated in 1×Perm/Wash Buffer (BD Biosciences, USA) for 10 min at RT. Cells were then resuspended in 1×Perm/Wash Buffer with primary antibodies (Supplementary Table S3) and incubated on ice for 1 h. Cells were washed twice with 1×Perm/Wash Buffer and analyzed using a Beckman Coulter DxFLEX flow cytometer. Analysis of the results was performed using FlowJo software.

**Western blot analysis**

Cells were harvested and washed twice with DPBS and then homogenized in RIPA buffer. Homogenates were incubated on ice for 20 min, centrifuged at 14,000 *g* for 20 min at 4°C, and supernatants were collected and used for immunoblotting analysis. The antibodies used are listed in Supplementary Table S3.

**Animal studies**

Immunodeficient SCID male mice, aged 8-10 weeks, were procured from Guangdong Medical Laboratory Animal Center. All experiments were conducted according to the experimental practices and standards approved by the Animal Welfare and Research Ethics Committee at Jinan University. For induction of diabetes, mice were administered 35 mg/kg streptozotocin via intraperitoneal injection for 5 days. For kidney capsule grafts, approximately 1×10^7^ hiPSC-differentiated cells were collected and injected under the renal capsule of diabetic mice. For glucose-induced C-peptide secretion, mice fasted overnight and serum was collected before and after intraperitoneal administration of 3g/kg D-glucose solution. Diabetic mice were randomized into three groups, and analysis was not blinded.
