# Supplementary material for: microRNA-181c-5p promotes the formation of insulin-producing cells from human induced pluripotent stem cells by targeting smad7 and TGIF2
Source: Cell Death Dis. 2020 Jun 15;11(6):462. doi: 10.1038/s41419-020-2668-9 (PMC7295798; doi:10.1038/s41419-020-2668-9)
Supplement: Supplementary file 2 — Supplementary Table S1 [file 41419_2020_2668_MOESM2_ESM.docx]

Supplementary Table S1. Primer sequences for mRNA analysis.

| Gene | Forward primer | Reverse primer |
| --- | --- | --- |
| OCT4 | GGGGTTCTATTTGGGAAGGTAT | TACTGGTTCGCTTTCTCTTTCG |
| NANOG | ATAACCTTGGCTGCCGTCTC | AGCCTCCCAATCCCAAACAA |
| CXCR4 | CTCCTCTTTGTCATCACGCTTCC | GGATGAGGACACTGCTGTAGAG |
| FOXA2 | CAAGGGCCAGAGTTCCACAA | CCTGCAACCAGACAGGGTAT |
| SOX17 | AAGGGCGAGTCCCGTATC | GTACTTGTAGTTGGGGTGGTCCT |
| HNF6 | AGGATAGAGGCAACACACCC | AGACTCCTCCTTCTTGCGTTC |
| PDX1 | CAGTTGAATGGGGCGGCAA | CAAGGTGGAGTGCTGTAGGAG |
| NKX6.1 | TGGCCTGTACCCCTCATCAA | GAATAGGCCAAACGAGCCCT |
| HNF4A | CTACATCAACGACCGCCAGT | ATCTGCTCGATCATCTGCCAG |
| SOX9 | GCTCTGGAGACTTCTGAACGA | CCGTTCTTCACCGACTTCCT |
| NKX2.2 | AGACGGGGTTTTCGGTCAAG | GACCGTGCAGGGAGTACTGAA |
| NGN3 | TTTTCTCCTTTGGGGCTGGG | AGGCGTCATCCTTTCTACCG |
| MAFA | CTTCAGCAAGGAGGAGGTCATC | CTCGTATTTCTCCTTGTACAGGTCC |
| NeuroD1 | ATGACCAAATCGTACAGCGAG | GTTCATGGCTTCGAGGTCGT |
| Insulin | GCAGCCTTTGTGAACCAACAC | CCCCGCACACTAGGTAGAGA |
| SST | GATCCGCGCCTAGAGTTTGA | AGTACTTGGCCAGTTCCTGC |
| GCG | CAGCACACTACCAGAAGACAGC | ACTGGTGAATGTGCCCTGTG |
| GATA4 | CGACACCCCAATCTCGATATG | GTTGCACAGATAGTGACCCGT |
| smad7 | ACTCCAGATACCCGATGGAT | AATTGAGCTGTCCGAGGCAA |
| TGIF2 | GGTGGGTCTAGCCAGGAGA | ATGGGCGACTCAGAAGTTGG |
| GAPDH | ACATCATCCCTGCCTCTACTG | ACCACCTGGTGCTCAGTGTA |
