# Supplementary material for: microRNA-181c-5p promotes the formation of insulin-producing cells from human induced pluripotent stem cells by targeting smad7 and TGIF2
Source: Cell Death Dis. 2020 Jun 15;11(6):462. doi: 10.1038/s41419-020-2668-9 (PMC7295798; doi:10.1038/s41419-020-2668-9)
Supplement: Supplementary file 3 — Supplementary Table S2 [file 41419_2020_2668_MOESM3_ESM.docx]

Supplementary Table S2. Primers and probe sequences for microRNA analysis.

| miRNA | Sequence (5’-3’) | Forward primer | RT primer |
| --- | --- | --- | --- |
| hsa-miR-375 | UUUGUUCGUUCGGCUCGCGUGA | CGGTTTGTTCGTTCGGCTC | GTGCAGGGTCCGAGGTCAGAGCCACCTGGGCAATTTTTTTTTTTCACGCG |
| hsa-miR-181c-5p | AACAUUCAACCUGUCGGUGAGU | TCGGAACATTCAACCTGTCG | GTGCAGGGTCCGAGGTCAGAGCCACCTGGGCAATTTTTTTTTTTACTCAC |
| cel-miR-54-5p | AGGAUAUGAGACGACGAGAACA | TCGGAGGATATGAGACGACG | GTGCAGGGTCCGAGGTCAGAGCCACCTGGGCAATTTTTTTTTTTGTTCTC |
| Universal reverse primer | CAGTGCAGGGTCCGAGGT |  |  |
| Universal Taqman probe | 56-FAM/CAGAGCCAC/ZEN/CTGGG  CAATTT/3IABkFQ |  |  |
