# Supplementary material for: microRNA-181c-5p promotes the formation of insulin-producing cells from human induced pluripotent stem cells by targeting smad7 and TGIF2
Source: Cell Death Dis. 2020 Jun 15;11(6):462. doi: 10.1038/s41419-020-2668-9 (PMC7295798; doi:10.1038/s41419-020-2668-9)
Supplement: Supplementary file 4 — Supplementary Table S3 [file 41419_2020_2668_MOESM4_ESM.docx]

Supplementary Table S3. Antibodies used.

| Antibody | Manufacturer | Dilution |
| --- | --- | --- |
| CXCR4-FITC | R&D System (FAB170F) | / |
| SOX17-APC | R&D System (IC1924A) | / |
| NKX6.1-Alexa Fluor 647 | BD Bioscience (563338) | / |
| PDX1- Alexa Fluor 488 | BD Bioscience (562274) | / |
| Insulin-APC | R&D System (IC1417A) | / |
| Goat IgG-APC Isotype Control | R&D System (IC108A) | / |
| Mouse IgG_2A_-FITC Isotype Control | R&D System (IC003F) | / |
| Mouse IgG_1_-Alexa Fluor 488 Isotype Control | R&D System (IC002R) | / |
| Mouse IgG_1_-Alexa Fluor 647 Isotype Control | R&D System (IC002G) | / |
| Rat IgG_2A_-APC Isotype Control | R&D System (F0130) | / |
| smad7 | R&D System (MAB2029) | 1:1000 |
| TGIF2 | Abcam (ab155948) | 1:1000 |
| p-smad2 | CST (18338) | 1:1000 |
| smad2 | CST (5339) | 1:1000 |
| p-smad3 | CST (9520) | 1:1000 |
| smad3 | CST (9523) | 1:1000 |
| β-Tubulin | TransGen Biotech (HC101) | 1:5000 |
